# Size- and morphology-dependent optical properties of ZnS:Al one-dimensional structures

**DOI:** 10.1007/s11051-015-3000-y

**Published:** 2015-04-18

**Authors:** Xianghua Zeng, Shunjun Yan, Jieya Cui, Hongfei Liu, Jing Dong, Weiwei Xia, Min Zhou, Haitao Chen

**Affiliations:** School of Physics Science and Technology & Institute of Optoelectronic Technology, Yangzhou University, Yangzhou, 225002 People’s Republic of China

**Keywords:** Al-doped ZnS, Morphology, Surface-enhanced Raman scattering, PL spectra

## Abstract

Typical morphology substrates can improve the efficiency of surface-enhanced Raman scattering; the need for SERS substrates of controlled morphology requires an extensive study. In this paper, one-dimensional ZnS:Al nanostructures with the width of approximately 300 nm and the length of tens um, and micro-scale structures with the width of several um and the length of tens um were synthesized via thermal evaporation on Au-coated silicon substrates and were used to study their size effects on Raman scattering and photoluminescent spectra. The photoluminescence spectra reveal the strongest green emission at a 5 at% Al source, which originates from the Al-dopant emission. The Raman spectra reveal that the size and morphology of the ZnS:Al nanowires greatly influences the Raman scattering, whereas the Al-dopant concentration has a lesser effect on the Raman scattering. The observed Raman scattering intensity of the saw-like ZnS:Al nanowires with the width of tens nm was eight times larger than that of the bulk sample. The enhanced Raman scattering can be regarded as multiple scattering and weak exciton—phonon coupling. The branched one-dimensional nanostructure can be used as an ideal substrate to enhance Raman scattering.

## Introduction

Surface-enhanced Raman scattering (SERS) spectroscopic methods are widely used to identify and provide structural information regarding molecular species at ultra-low concentrations (Kneipp et al. 1997, 2006; Nelayah et al. 2007). The efficiency of a SERS substrate is described by its enhancement factor (EF), which is quantitatively related to the ratio between the enhanced electric fields at the metallic surface where the Raman scattering molecule is located and the incident field away from the surface. To improve SERS, different types of techniques have been used to provide more focused “hotspots” and enhance large electromagnetic fields. Furthermore, studies have shown that SERS relies strongly on controlling the nano- and micro-scale morphology of metal nanostructured SERS substrates (Farcau and Astilean 2014); as the particles are placed in a highly ordered regular array the long-range photonic interactions can produce sharp resonances (Genov et al. 2004), at the same time nanoparticles exhibit shape anisotropy and posses multiple resonances. Therefore, the localized surface plasmon resonance has size, shape, material, and interparticle spacing effects. For example, designing 3D mesoscopic multipetal flowers assembled by metallic nanoparticles as SERS substrates to improve surface-enhanced Raman spectroscopy has been reported (Jung et al. 2014); using holographic laser illumination of a silver nanohole array, the authors observed dynamic placement of locally enhanced plasmonic fields (Ertsgaard et al. 2014).

Although there have been some discussions regarding enhanced Raman scattering, the mechanism behind it is still up for debate. Typically, the main mechanism of the enhanced Raman scattering is considered to be a combination of two effects: an electromagnetic effect as a result of an increase in the strength of the electric field at the molecule and a resonance-like charge transfer effect. These locally enhanced fields are called plasmonic “hotspots”, which can produce up to 14–15 orders of magnitude enhancement of the surface plasmon compared to normal Raman scattering caused by the creation of an electromagnetic resonator on the surface of the nanomaterial (Camden et al. 2008). The charge transfer enhancement is typically less than two orders of magnitude (Haynes et al. 2005). In Kneipp et al. 1997, seven orders of SERS enhancement and multiresonance features in the entire visible frequency range were achieved by plasmonic hot-spot engineering by increasing the number of petals from four to eight. By studying the effects of the surface roughness, strong Raman scattering effects (Reyes et al. 2010) were found, which were ascribed to the polarization-dependent behavior of CdS. Furthermore, the enhanced Raman scattering and enhanced exciton-LO-phonon coupling observed in the CdS ripple-like MBs (Zeng et al. 2014) were ascribed to larger exciton polarizations caused by surface defects via the Fröhlich interaction. The enhanced Raman scattering observed for ZnSe nanoparticles was ascribed to an aggregation of the ZnSe particles, resulting in dipolar interactions and improvement of the Fröhlich interaction (Li et al. 2010). Recently, a remarkable increase in the Raman sensitivity of optimized Si nanowire arrays was determined to result from surface multiple scattering, characterized by a large spatial extension (approximately fifty nm) (Bontempi et al. 2014). By studying the different concentrations of Mn doped into the CdS nanoparticles, the researchers found (Zhao et al. 2013) that the enhancement in the 1LO-phonon intensity was caused by the interstitial Mn dopants, which decrease the NC surface deformation potential because of the small dielectric constant of the metal, resulting in enhanced coupling between the LO phonon and the surface plasmon. Some people (Lin et al. 2004) ascribed the selective enhancement of Raman intensity to the presence of a gold catalyst, which was responsible for plasmon scattering at the ZnS/Au interface; however, the changes in the Raman intensity were not obvious for the ZnS powders in the absence of the gold catalyst.

There are many discussions on SERS, but most of them are based on the optical near-field intensity enhancement on the metallic (plasmonic) substrate; the mechanism of the enhanced Raman scattering from low-dimensional nonmetallic materials should be studied. Unlike photolithography (Perney et al. 2006), etching (Alvarez-Puebla et al. 2007), and spark discharge (Jung et al. 2014) methods which were used to prepare the SERS substrates, in this paper the simple thermal evaporation method was used to prepare one-dimensional (1D) periodic structures, that is, saw-like and firry leaf-like ZnS:Al nanowires, as well as normal and banana leaf-like ZnS:Al microbelts. Their lattice structures and dopant concentrations are discussed from XRD images and the Rietveld method. Raman scattering and photoluminescence measurements were used to study the effects of the sizes and morphologies on the optical properties, where the morphology-dependent Raman scattering was observed, at the same time, the mechanism of the enhanced Raman scattering was discussed.

## Experimental section

ZnS:Al nanowires were deposited on Au-coated Si substrates in a horizontal tube furnace via thermal evaporation. The zinc sulfide powder [ZnS (99.999 %)] and aluminum powder [Al (99.99 %)] source materials were obtained from Aladdin (Shanghai, China) and Sinopharm Chemical Reagent Co, Ltd. (Shanghai, China), respectively. First, 0.3 g ZnS powders and different contents of Al powder were placed separately into two identical quartz boats, which were then placed at the center of the heating zone. Then, one Si (100) substrate coated with an Au film was placed obliquely downstream of the Al powder and subsequently ZnS powders at a distance of 11 cm. Before heating, the system was purged with 522 standard cubic centimeter per minute (sccm) of high-purity argon (Ar, 99.999 %) for 30 min. Then, the pressure was reduced to 7.5 × 10^−2^ Torr for the duration of the reaction. Next, the furnace was heated to the desired temperature of 1050 °C at a heating rate of 10 °C/min and was maintained at this temperature for 30 min with a constant Ar flow of 50 sccm. After the system was cooled to room temperature, a white-colored, wool-like product was deposited onto the silicon substrate. Finally, four types of ZnS:Al samples were obtained with atomic ratios of Al to ZnS equal to 3, 5, 7, and 10 % in the two initial quartz boats, which were labeled as #A1, #A2, #B1, and #B2, respectively.

The as-synthesized products were characterized via X-ray diffraction (XRD-7000, Shimadzu) using Cu K*α* radiation (*λ* = 0.15406 nm). The XRD data were collected in the 2θ range of 10°–65° using a continuous scanning method at a scanning speed of 10° (2θ)/min. The morphology and microstructure of each sample were observed using a field-emission scanning electron microscope (FESEM, s-4800II, Hitachi) equipped with an X-ray energy dispersive spectrometer (EDS) and a high-resolution transmission electron microscope (HRTEM, Tecnai F30, FEI). The absorption measurements were performed using a UV–Vis–NIR spectrophotometer (UV–Vis, Cary-5000, Varian) with an integrating sphere. The Raman measurements were performed using a Renishaw In Via equipment. The photoluminescence (PL) measurements were performed on a FLS920 instrument.

## Results and discussion

### Structures and morphologies of the ZnS:Al samples

**Fig. 1:**
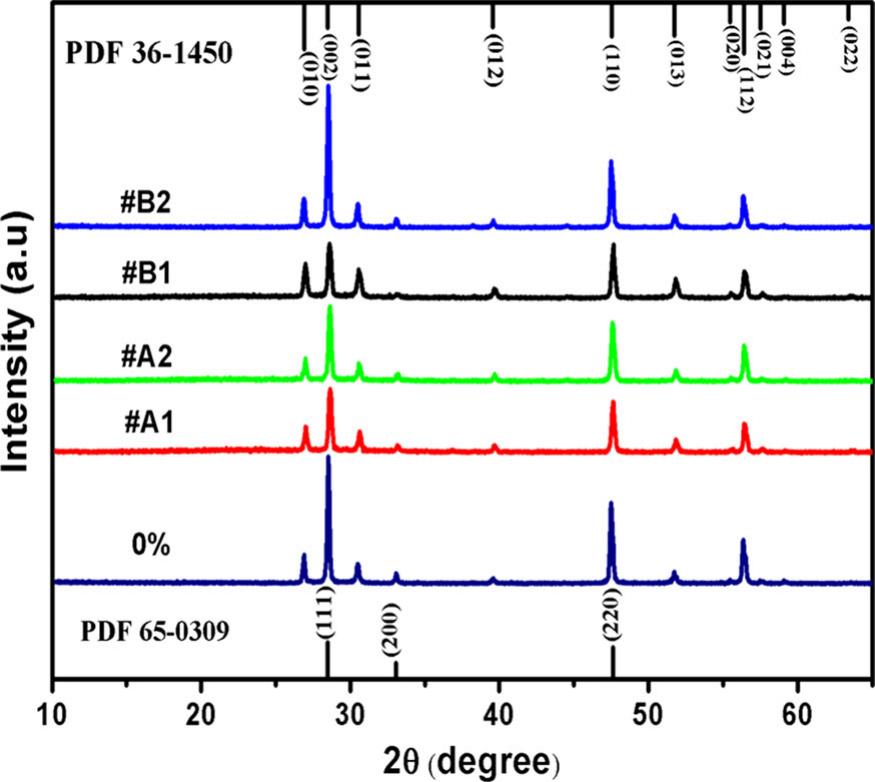
XRD patterns of the undoped ZnS and ZnS doped with Al at the atomic ratios of Al to ZnS sources equal to 3 % (#A1), 5 % (#A2), 7 % (#B1), and 10 % (#B2) from the two initial quartz boats. Both the wurtzite structure (JCPDS 36-1450) and zinc blende structure (JCPDS 65-0309) are listed for reference

**Fig. 2:**
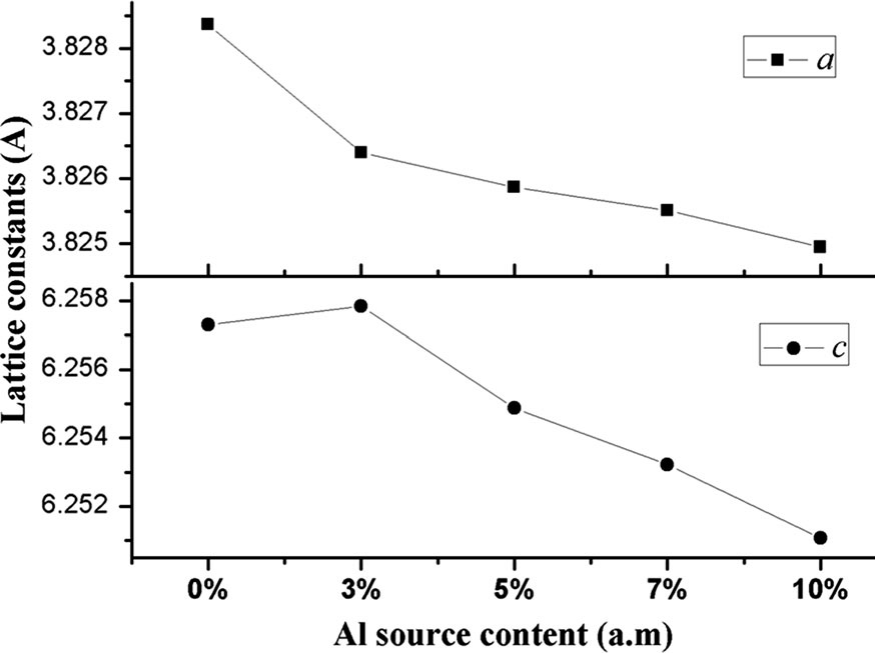
Lattice constants for the undoped sample and samples #A1, #A2, #B1, and #B2

The XRD patterns of the ZnS:Al samples are shown in Fig. 1. To determine the unit cell parameters, the XRD patterns were analyzed by a simple Pawley refinement method using the Topas Academic V5.0 software. The XRD images show that nearly all of the peaks agree with the ZnS wurtzite structure (JCPDS 36-1450), except for a small peak at 32.8°. Furthermore, the ZnS NW exhibits a space group of P63 mc, in which the main peaks at 2Θ = 28.52, 47.50, 26.89, 30.50, and 56.33° correspond to the directions of the (002), (110), (010), (011), and (112) planes, and the small peak at 32.8° agrees well with the (200) plane of ZnS zinc blende structure (JCPDS 65-0309). Because the (002) and (110) planes of the ZnS wurtzite structure overlap with the (111) and (022) planes of the ZnS zinc blende, the structures of the ZnS:Al samples can be regarded as a mixture of wurtzite and zinc blende structures with a dominant wurtzite ZnS. Additionally, the lattice constants were obtained from the Rietveld Refinement Treatment, as shown in Table 1. For the lattice constants, *a* = *b* = 3.8284 and *c* = 6.2573 Å were found for the undoped NWs; and *a* = 3.8264, 3.8259, 3.8255, 3.8250 Å and *c* = 6.2578, 6.2549, 6.2532, 6.2511 Å were found for #A1, #A2, #B1, and #B2, respectively. Compared to the undoped ZnS, the lattice constant *a* contracted by 0.197, 0.25, 0.286, and 0.342 %, and the constant *c* contracted by −0.054, 0.243, 0.408, and 0.623 % were found for samples #A1, #A2, #B1, and #B2, respectively. Except for the constant *c* of sample #A1, the lattice constants *a* and *c* decreased with increasing content of the Al source. A gradual decrease in the lattice parameters indicates that an increasing number of Al atoms are successfully incorporated into the ZnS host as the Al source content increases, which is consistent with the relations of Vegard’s law, as shown in Fig. 2. With the increase of Al^3+^ ions, the lattice parameters of the ZnS:Al host decreases because the ionic radius of Al^3+^ (54 pm) is smaller than that of Zn^2+^ (74 pm). Furthermore, an abnormal increase of the lattice constant *c* of sample #A1 can be regarded as the surface tensile induced by its typical morphology. Because the Al-dopant concentration is very low when using the thermal evaporation method and is hard to identify in the EDS because of its small quantities, the XRD Rietveld Refinement is used to provide evidence for the incorporation of atoms into the ZnS host. The quantitative elemental analysis from the EDS spectra for the ZnS:Al samples indicate that Zn and S are major elements; however, the S/Zn atomic ratio is less than one, indicating that sulfide vacancies are present in the ZnS:Al samples.

**Table 1:** Lattice parameters for different samples

Source Al content (%)	Lattice constant a	Lattice constant c	Contraction rate of a (%)	Contraction rate of c (%)
0	3.82837	6.2573	–	–
3 (#A1)	3.8264	6.25784	0.197	−0.054
5 (#A2)	3.82587	6.25487	0.25	0.243
7 (#B1)	3.82551	6.25322	0.286	0.408
10 (#B2)	3.82495	6.25107	0.342	0.623

**Fig. 3:**
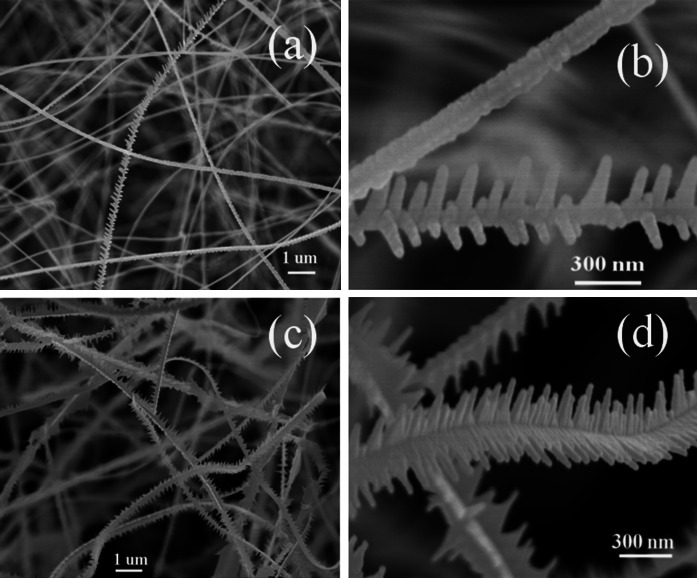
SEM and FESEM images of samples #A1 (**a**, **b**) and #A2 (**c**, **d**)

**Fig. 4:**
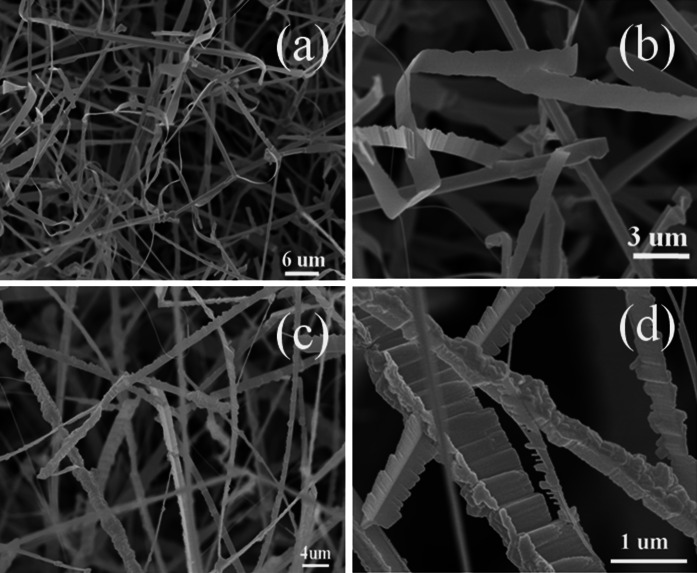
SEM and FESEM images of samples #B1 (**a**, **b**) and #B2 (**c**, **d**)

The morphologies and compositions of the products were characterized via FESEM, as shown in Figs. 3 and 4. Figure 3a, b represent the SEM images of sample #A1, whereas Fig. 3c, d represent the SEM images of sample #A2. For sample #A1, some of nanowires have a main bough and many needle-like twigs distributed alternatively along each side. The needle-like twig has a diameter of tens to one hundred nanometers at the base in contact with the bough, and the diameter of the tip of the needle is several to a few tens of nanometers. Additionally, there is a several to tens of nm separation between two neighboring needles, making the structure resemble a pine leaf. And some nanowires have a saw-like structure with a width of the order of tens of nanometers. For sample #A2, each NW resembles a firry leaf with a length ranging from tens to several hundred nanometers and a width of several hundred nm, but without the main bough and twigs, as shown in Fig. 3c, d. From Fig. 3 a–d one can find that the size of sample #A2 is larger than that of sample #A1. Sample #B1 has a microbelt shape with a width of 1–3 micrometers and a length in the tens of micrometers, as shown in Fig. 4c. Sample #B2 is shaped like a banana leaf and exhibits the same size as sample #B1 but with a greater thickness, as shown in Fig. 4d. Sample #B2 more closely resembles the bulk material.

### Absorption and PL spectra of the ZnS:Al samples

**Fig. 5:**
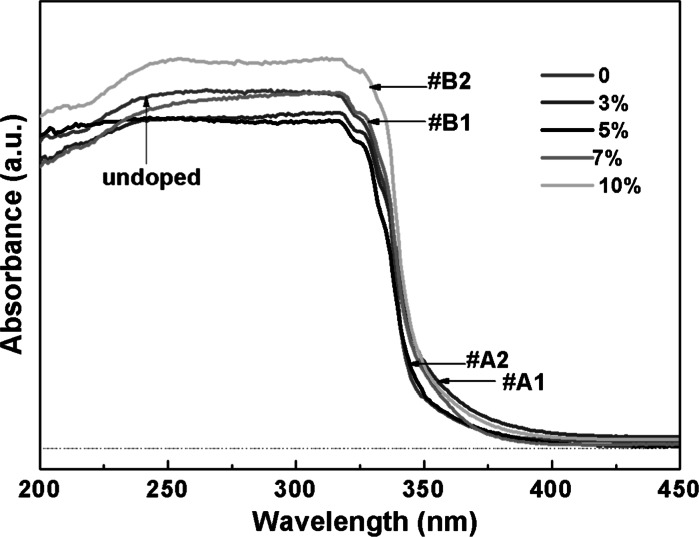
Absorption spectra of undoped ZnS and Al-doped ZnS samples #A1, #A2, #B1, #B2

UV–Vis absorption spectra were obtained by measuring the optical absorption spectra on a UV–Vis spectrophotometer (Cary-5000) with an integrating sphere to guarantee a better measurement result, as illustrated in Fig. 5. The absorption spectra of samples #A1, #A2, #B1, #B2, and undoped ZnS semiconductor can be extracted from the formula (*αhv*) = *c*(*hv* − *E*_g_)^1/2^, where *c* is a constant, *E*_g_ is the band gap, and the direct band gaps estimated from a plot of (*αhv*)^2^ versus the photon energy *hv* were equal to 3.626, 3.586, 3.612, 3.608, and 3.611 for undoped, #A1, #A2, #B1, and #B2, respectively. The band gap values show that the Al-doped samples have a slightly smaller band gap in comparison to the undoped sample, indicating that some Al atoms have been doped into the ZnS hosts. Sample #A1, with an atomic ratio of Al to Zn source equal to 3 at%, has the lowest band gap, which can be explained by an increase of surface defects in addition to the Al dopant because of the relatively smaller size and the large surface to volume ratio.

**Fig. 6:**
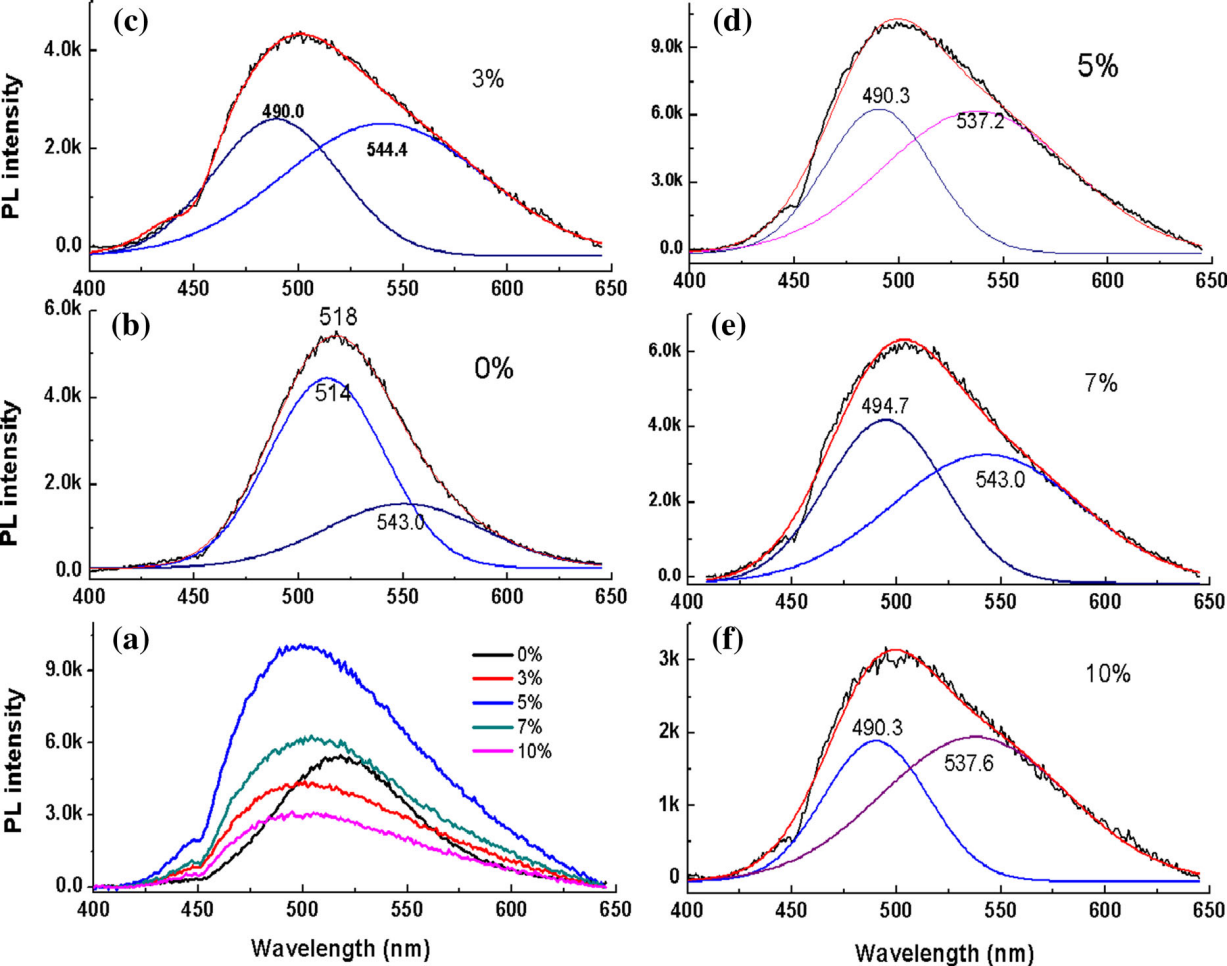
Room-temperature PL spectra of undoped ZnS and Al-doped ZnS samples (**a**); deconvolutions with Gaussian fitting for undoped ZnS (**b**), #A1 (**c**), #A2 (**d**), #B1 (**e**), and #B2 (**f**)

The PL properties of the undoped and Al-doped nano- and micro-scale ZnS structures were investigated via PL excitation at 325 nm to further assess their doping and structure qualities, as shown in Fig. 6. For the undoped ZnS structure, a slightly asymmetric emission at 518 nm was observed, and the Gaussian fitting revealed a dominant emission at 514 nm with a weak emission at 550 nm. The peak at approximately 514 nm has previously been attributed to the S vacancies in ZnS nanowires (Zhang et al. 2005), or ascribed to the peak at 518 nm representing the transitions of electrons from S vacancy states to zinc vacancy states (Kumar et al. 2006). The peak at 550 nm originates from bulk defects (Lei et al. 2011). For the Al-doped ZnS, a broad and asymmetric emission spectrum at approximately 500 nm was observed, and the deconvoluted peaks at ~490 and ~540 nm can be identified using a Gaussian fitting, as shown in Fig. 6c–f. For the four Al-doped ZnS nanostructures, sample #A2 (with 5 at% Al content in the source) exhibited a stronger emission than the other three samples, which is consistent with other reports, in which the highest luminescence intensity corresponded to the Al-dopant concentration of 6 at% for the ZnS:Al films (Prathap et al. 2009). Herein, the enhanced emission at 493 nm can be ascribed to the Al-doped ZnS nanoparticles, presumably from the donor–acceptor pair (DAP) transition (Nagamani et al. 2012); the observation of which is considered to be an obvious evidence for the substitution of Al in the host lattice in ZnS:Al nanoparticles (Reddy et al. 2014). The green band at 530 nm may be related to some native defects of pure ZnS (Sotillo et al. 2012, 2014), such as zinc vacancies (Mitsui et al. 1996, Chen et al. 2011) or S vacancies (Ye et al. 2004; Jiang et al. 2012). A decrease in the emission intensity at 500 nm with Al-dopant concentration can be explained by the formation of defect complexes (Al donors) that induces the structural disorder caused by an increase in non-radiative recombination (Prathap et al. 2009).

### Raman spectra of ZnS:Al nanostructures

**Fig. 7:**
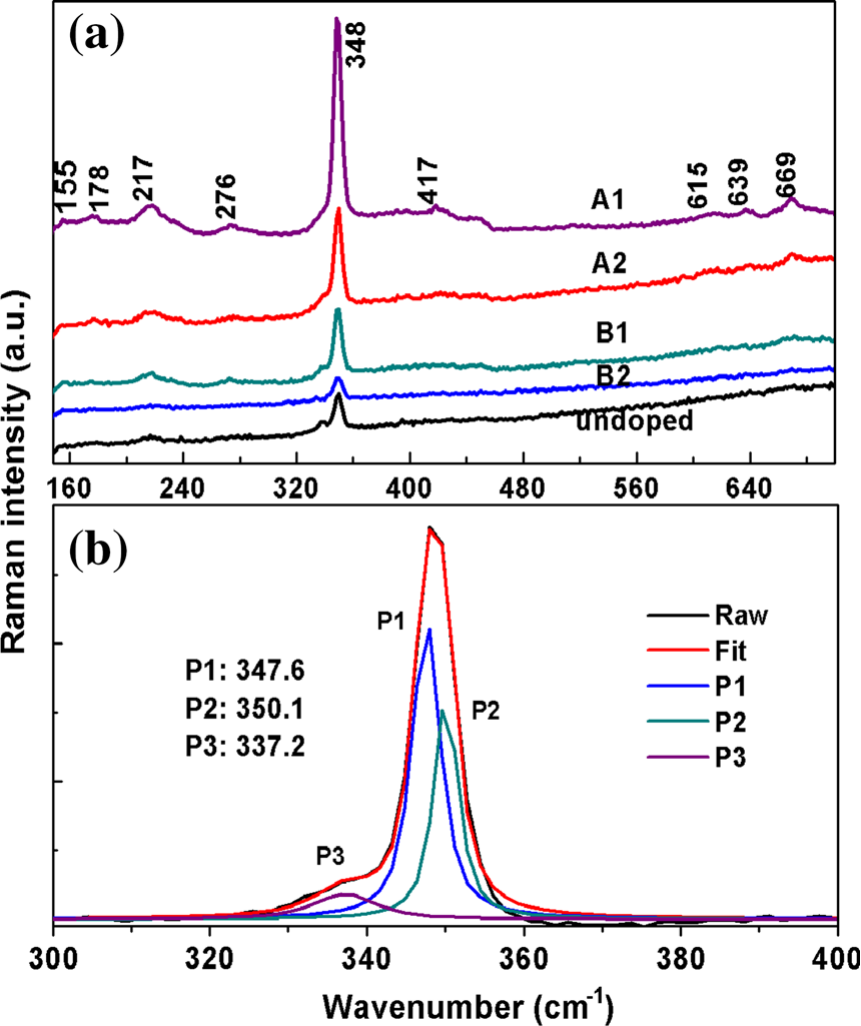
**a** Raman spectra for samples #A1, #A2, #B1, and #B2; **b** Raman decomposition at 347 cm^−1^ fitted with three Lorentzian curves for sample #A1; the peaks at 347.60, 350.14, and 337.2 cm^−1^ are labeled as P1, P2, and P3, respectively

Raman spectral measurements of the samples were conducted using an excitation wavelength of 532 nm with a 3 % incident laser light power of 300 mW to study the effects of size and morphology on the Raman scattering spectra. The room-temperature Raman spectra for undoped and different Al-doped ZnS materials are shown in Fig. 7a. For all of the samples, a sharp peak at 348 cm^−1^ was clearly observed. For the sample #A1 in Fig. 7a, the following peaks were clearly identified: a mode at ~217 cm^−1^ corresponding to the second-order longitudinal acoustic (2LA) phonon in ZnS (Cheng et al. 2009); a peak at 414 cm^−1^ associated with the LO+TA phonon mode; two peaks at 639 and 669 cm^−1^ attributed to the second-order optical phonon LO+TO and 2LO modes (Lin et al. 2004; Krol et al. 1988), respectively; two small peaks at 155 and 178 cm^−1^ assigned to disorder-activated second-order acoustic phonons (Scocioreanu et al. 2012); and a broad peak at 276 cm^−1^, which consists of the unresolved A1(TO) and E1(TO) phonon modes at 273.3 cm^−1^ and the E2(TO) phonon mode at 286.0 cm^−1^, as discussed in (Radhu and Vijayan 2011; Adu et al. 2006). Furthermore, for all samples, the peak at 348 cm^−1^ can be fitted using three Lorentzian curves, as shown in Fig. 7b, where the decomposition of the peak at 348 cm^−1^ from sample #A1 exhibits three peaks located at 347.60 (called as P1), 350.14 (P2), and 337.2 cm^−1^ (P3) with full-width-half-maxima (FWHM) of 4.377, 3.733, and 9.786 cm^−1^, respectively. The peaks at 347.60 (P1) and 350.14 cm^−1^ (P2) can be regarded as the E_1_(LO) and A_1_(LO) vibration modes, in which the E_1_ (LO) mode results from the lateral morphology of the ZnS nanowires parallel to the c-axis, as discussed in (Lu et al. 2005), whereas the peak at 337.16 cm^−1^ is assigned to a surface phonon mode (Kim et al. 2012). For the four samples, an identical decomposition was performed with nearly the same magnitude of the E_1_ (LO) and A_1_ (LO) phonons and their FWHMs. As shown in Table 2, the surface phonon was also found to be approximately 337 cm^−1^, except for sample #B2 in which the value was red-shifted approximately 4 cm^−1^ and the FWHW was larger because of the disappearance of the surface effects for sample #B2. Compared to the Raman spectrum of bulk hexagonal ZnS (LO: 352 cm^−1^) (Brafman and Mitra^1968^; Nilsen 1969), the peaks of the first-order LO phonons from these ZnS nanowires exhibit a shift toward lower energy.

**Table 2:** Deconvolutions of the Raman peak at 348 cm^−1^ with Lorentzian curves

Sample	Peak position (cm^−1^)	Integrated area	FWHM (cm^−1^)
#A1	347.595	55,926.9	4.377
	350.136	35,563	3.733
	337.159	10,619.16	9.786
#A2	347.529	19,449.05	4.073
	350.138	20,608.61	3.6
	338.083	15,641.78	12.773
#B1	350.258	11,049.53	3.439
	347.717	17,371.09	4.255
	337.759	9086.667	10.521
#B2	350.676	3056.963	3.585
	347.855	8199.131	5.961
	333.435	4480.878	19.426
Undoped	350.264	6228.291	2.983
	347.555	7377.402	3.976
	336.972	9621.145	11.435

To study the changes of the Raman scattering intensity, we selected sample #B2 as a reference and determined that the ratios of the 1LO-integrated areas of #A1, #A2, and #B1 to that of #B2 are equal to 8.13, 3.56, and 2.52, respectively, indicating that sample #A1 exhibits greatly enhanced Raman scattering. Comparing samples #A1 and #A2, the greatest difference is in their size and morphological structures. For sample #A1, the pine leaf-like nanowires grow along a main bough with many needle-like twigs alternatively grown along the two sides. Additionally, there is a several to tens of nanometers separation between two neighboring needles, resulting that the diameter for the main bough and the twig is only several tens of nanometer. Even for the saw-like structure nanowires in sample #A1, the diameter is only approximately 100 nm. For sample #A2, although many ZnS:Al nanostructures grow along both sides of the z-direction in a firry leaf-like nanowire, there is no independent nanopattern; their sizes are far more larger than that of sample #A1, thus the surface to volume ratio decreases. Therefore, the greater enhancement of the Raman scattering observed from sample #A1 is mainly attributed to its typical morphology and small size of nanopatterns. The phenomenon of the effect of morphology on the Raman scattering was observed in (Fasolato et al. 2014), where the enhanced Raman spectra were attributed to the different cluster morphologies rather than to the different Ag nanoparticle diameters. Additionally, optimized high-density Si nanowire arrays have demonstrated a remarkable increase in Raman sensitivity compared to reference planar samples, which was explained as surface multiple scattering (Bontempi et al. 2014). Comparing the samples #A2, #B1, and #B2, the 1LO-integrated area gradually decreases, implying that as the Al-dopant concentration increases, the Raman scattering intensity decreases. This observation contradicts the results (Zhao et al. 2013) of Mn-doped CdS nanoparticles, which showed that the Raman scattering intensity increased with increasing dopant content before decreasing.

Actually, surface-enhanced Raman scattering (SERS) will take place when the energy of the incident laser light is close to the surface plasmon energy in noble metal nanoclusters which are intentionally introduced in NC arrays, or the energy of the incident laser light matches that of interband electronic transitions in the NCs. For nanostructure materials both quantum effects and surface effects are more important, and size- and shape-dependent Raman effects will be more obvious. Theoretically, the calculations (Zenidaka et al. 2011) of the SERS based on the optical near-field intensity enhancement on the metallic (plasmonic) and the nonmetallic (Mie scattering) nanostructured substrates with two-dimensional (2D) periodic nanohole arrays indicated that both the inter-void distance and the void diameter influence the optical intensity enhancement. Experimentally, the sensitivity of the field enhancement in the hot spots to the distance of the particles, as well as the frequency and polarization of the excitation laser have been reported (KNEIPP et al. 2006). In our studies, the enhanced Raman scattering from interband electronic transitions can be excluded since all the PL spectra centered at approximately 500 nm, and the strongest PL intensity is not for #A1 but for #A2. As reported in (Hu et al. 2013), the coupling strength of the exciton—phonon increases with increasing lateral size, which will result in the reduction of the Raman scattering. Therefore, the greatly enhanced Raman scattering for sample #A1 can be explained by the large surface multiple scattering and a surface polarization effect.

## Conclusion

In this paper, different morphological ZnS:Al nano- and micro-scale structures were synthesized using a thermal evaporation method, and the lattice constants were analyzed by a simple Pawley refinement method using the Topas Academic V5.0 software. The results showed that the lattice constants decreased with increasing Al source contents, indicating that more Al atoms were doped into the ZnS host. The PL spectra showed that there is a slightly asymmetric emission at 518 nm for the undoped ZnS nanostructure, which is composed of a dominant emission at 514 nm and a weak emission at 550 nm using a Gaussian fitting, corresponding to the S vacancies and the bulk defects, respectively. For the Al-doped ZnS, the broad, asymmetric emission observed at 500 nm can be deconvoluted into two peaks at ~490 and ~540 nm using a Gaussian fitting, which correspond to the Al-doped ZnS and the native defects of pure ZnS, respectively. The strongest green emission corresponds to the Al-doped sample at 5 at% Al source. Finally, the Raman spectra showed that the size and the morphology have great influence on the Raman scattering, and that ZnS:Al one-dimensional branched nanowires with a small size demonstrate the greatest enhancement in Raman scattering. Compared to the size and morphology, the Al-dopant concentration had minimal effect on the Raman scattering, i.e., the Raman scattering intensity slightly decreases with an increase in Al-dopant concentration. At the same time, the enhanced Raman scattering from interband electronic transitions was not obvious. The enhanced Raman scattering can be regarded as multiple scattering and weak exciton—phonon coupling. The branched one-dimensional nanostrcuture can be used as an ideal substrate to enhance Raman scattering. But more detailed physics mechanism need to be further discussed. Our studies will help to obtain an efficient SERS substrate to improve surface-enhanced Raman spectroscopy.
